# Supplementary material for: Testing for heavy metals in drinking water collected from Dog Aging Project participants
Source: PLOS Water. Author manuscript; Available in PMC 2025 Sep 26. (PMC12463316; doi:10.1371/journal.pwat.0000296)
Supplement: S2 Text. Water sampling kit instructions. [file NIHMS2106642-supplement-S2_Text__Water_sampling_kit_instructions_.pdf]

DOG AGING PROJECT WATER SAMPLING KIT
Read these instructions the day before filling your bottle!

- Step 0. Plan ahead. It is ***very*** important to collect water **after allowing it to sit in your pipe for at least 6 hours**. To do so easily, water should be collected first thing in the morning (after no one uses the water from that tap overnight) *from your dog's primary drinking water source*.

- Step 1. After the water was not used for at least 6 hours, open the kit and remove the plastic cap from the bottle.

- Step 2. With the 250 mL collection bottle held under the water tap, open the cold water tap and fill the bottle at high flow (as if you were filling a pitcher of water).

- Step 3. Turn off the flow, put the cap back on the bottle, and firmly tighten the cap.

- Step 4. Complete the sample label as instructed above.

- Step 5. Place the bottle into the Ziplock bag that is provided. Seal the Ziplock bag and place the bag (with bottle inside) into the box that is provided.

- Step 6. Tape the box shut, apply the pre-paid return shipping label, and drop at any UPS collection site.

- Call the Ruple Lab at 540-231-0342 or email ruplelab@gmail.com if you have any questions regarding these instructions.
