# Supplementary material for: Testing for heavy metals in drinking water collected from Dog Aging Project participants
Source: PLOS Water. Author manuscript; Available in PMC 2025 Sep 26. (PMC12463316; doi:10.1371/journal.pwat.0000296)
Supplement: S3 Text. Sample data return. [file NIHMS2106642-supplement-S3_Text__Sample_data_return_.pdf]

Dear {Participant},

Thank you for participating in our water quality study for the Dog Aging Project! We are so grateful for your help with this research and wanted to provide you with the results of our analysis.

After we received your sample, we analyzed [Dog's name]'s drinking water for the presence of 28 different elements. Many of these elements are commonly found in drinking water and are not known to cause any health issues. However, eight of the elements are heavy metals that are listed by the Environmental Protection Agency (EPA) as having Maximum Contaminant Level Goals (MCLGs).

If one of these metals occurs in your water at a level below the MCLGs, the EPA considers the water safe to drink. The EPA is concerned about levels above the MCLGs and provides additional information and precautionary steps to consumers like yourself at this link: National Primary Drinking Water Regulations | US EPA.

Below, we display the results for this study for the eight heavy metals in parts per billion (ppb). In the table, we present two numbers for each heavy metal:

- *MCLG: EPA Maximum Contaminant Level Goal for each metal.*
- *YOUR RESULT: the level from the water sample you submitted.*

The MCLGs for each metal are depicted as the black vertical line. Your result is the small blue line.

		MCLG	**YOUR RESULT**
Arsenic	0 — 20 ppb	10	0
Barium	0 — 4000 ppb	2000	2.5
Cadmium	0 — 10 ppb	5	0
Chromium	0 — 200 ppb	100	0
Copper	0 — 2600 ppb	1300	1415.4*
Lead	0 — 30 ppb	15	0.9
Selenium	0 — 100 ppb	50	0
Uranium	0 — 60 ppb	30	0

MCLG **YOUR RESULT**

At least one of your values (denoted in red with an asterisk) is to the right of the MCLG line. We recommend that you visit National Primary Drinking Water Regulations | US EPA for more information.

We also tested 20 other elements that are not regulated by the EPA. Not much is known about the effects of these other 20 elements in ______'s drinking water. This current lack of information is part of why we are studying this (with your help)! The table below lists each element and displays the following results in either parts per million (ppm) or parts per billion (ppb). In the absence of known MCLGs for these elements, we have also provided the quartile your result is in compared to other study participants to allow for some comparison.

	Quartile				YOUR RESULT		Quartile				YOUR RESULT
	1	2	3	4			1	2	3	4	
Aluminum	*				0.0 ppb	Potassium	*				0.0 ppb
Calcium	*				240.0 ppb	Silicon			*		11704.1 ppb
Chlorine	*				3.1 ppm	Silver	*				0.0 ppb
Cobalt	*				0.0 ppb	Sodium			*		38687.6 ppb
Iron	*				0.0 ppb	Strontium	*				0.0 ppb
Lithium				*	11.2 ppb	Sulfur		*			10.6 ppm
Magnesium		*			228.0 ppb	Tin	*				0.0 ppb
Manganese				*	5.5 ppb	Titanium				*	1.3 ppb
Nickel				*	5.8 ppb	Vanadium	*				0.0 ppb
Phosphorus		*			5.4 ppb	Zinc				*	470.5 ppb

Quartile 1: Your result is in the lowest 25% of study participants.
Quartile 2: Your result is higher than 25% and lower than 50% of study participants.
Quartile 3: Your result is higher than 50% and lower than 25% of study participants.
Quartile 4: Your result is in the highest 75% of study participants.

If you have any additional questions about your results, consider reaching out to your local community health department. If you have questions about this study, please email ruplelab@gmail.com. Thank you again for your participation in this important study!

Sincerely,

The team at the Dog Aging Project

Dog Aging Project

Longer, healthier lives. Together.
